# Ocular siderosis: a misdiagnosed cause of visual loss due to ferrous intraocular foreign bodies—epidemiology, pathogenesis, clinical signs, imaging and available treatment options

**DOI:** 10.1007/s10633-020-09792-x

**Published:** 2020-09-19

**Authors:** Giamberto Casini, Francesco Sartini, Pasquale Loiudice, Gabriella Benini, Martina Menchini

**Affiliations:** 1 Ophthalmology, Department of Surgical, Medical, Molecular Pathology and of the Critical Area, University of Pisa, Via Savi, 10, 56126 Pisa, Italy; 2 Ophthalmology, Hospital of Leghorn, Leghorn, Italy

**Keywords:** Ocular siderosis, Intraocular foreign body, Electroretinogram, Penetrating ocular trauma

## Abstract

**Purpose:**

The purpose of this paper is to provide a meaningful literature review about the epidemiology, pathogenesis, clinical signs, imaging and treatment of ocular siderosis (OS).

**Methods:**

A computerized search from inception up to March 2020 of the online electronic database PubMed was performed using the following search strings: “ocular siderosis” and “siderosis bulbi”. The reference list in each article was analysed for additional relevant publications.

**Results:**

OS is an uncommon cause of visual loss due to a retained ferrous intraocular foreign body (IOFB). It may develop from 18 days to years after a penetrating trauma that usually occurs during hammering. On average, patients are 22–25 years old, and the vast majority are male. The most common cause of OS development is delayed presentation by the patient or missed diagnosis of IOFB after trauma. The pathophysiology is not fully understood; nevertheless, iron deposition causes hydroxyl radical formation, which damages photoreceptors and retinal pigment epithelium. Moreover, iron damages retinal vessels with consequent inner retinal layers degeneration. The most frequent signs are iris heterochromia, pupillary mydriasis, cataract development and retinal arteriolar narrowing with pigmentary retinal degeneration. Electroretinogram signs, in particular, *b*-wave amplitude reduction, arise earlier than clinical signs. Orbital CT scans and ultrasonography play an essential role in detecting IOFBs. Treatment depends on the IOFB location and OS development. However, it is crucial to remove the IOFB after OS development because visual acuity and clinical signs may improve. Anterior segment IOFBs can be dislodged using an intraocular magnet (IOM) or forceps through limbal paracentesis. In contrast, posterior segment IOFBs require a pars plana vitrectomy and IOM or forceps to be removed through an enlarged sclerotomy or the limbus.

**Conclusion:**

Recommending the usage of protective glasses and spreading knowledge about OS may further benefit patient care.

## Introduction

Ocular siderosis (OS) is an uncommon cause of visual loss that was first described in 1890 by Bunge with the term “siderosis bulbi” [1]. It is due to a retained ferrous intraocular foreign body (IOFB) that causes iron deposition in ocular tissues [2, 3]. Rarely, siderosis can be caused by an IOFB presumed to not contain free iron, such as stone or steel, or by vitreous haemorrhage [4, 5].

Therefore, IOFB presence must be excluded in cases of penetrating ocular injury, especially with a history of high-velocity metallic injury [6]. A complete ophthalmic evaluation and imaging must be carried out to prevent OS development; nevertheless, IOFB localization can sometimes be difficult, in particular, if hyphema, cataract or vitreous haemorrhage occurs [6].

## Methods

**Fig. 1:**
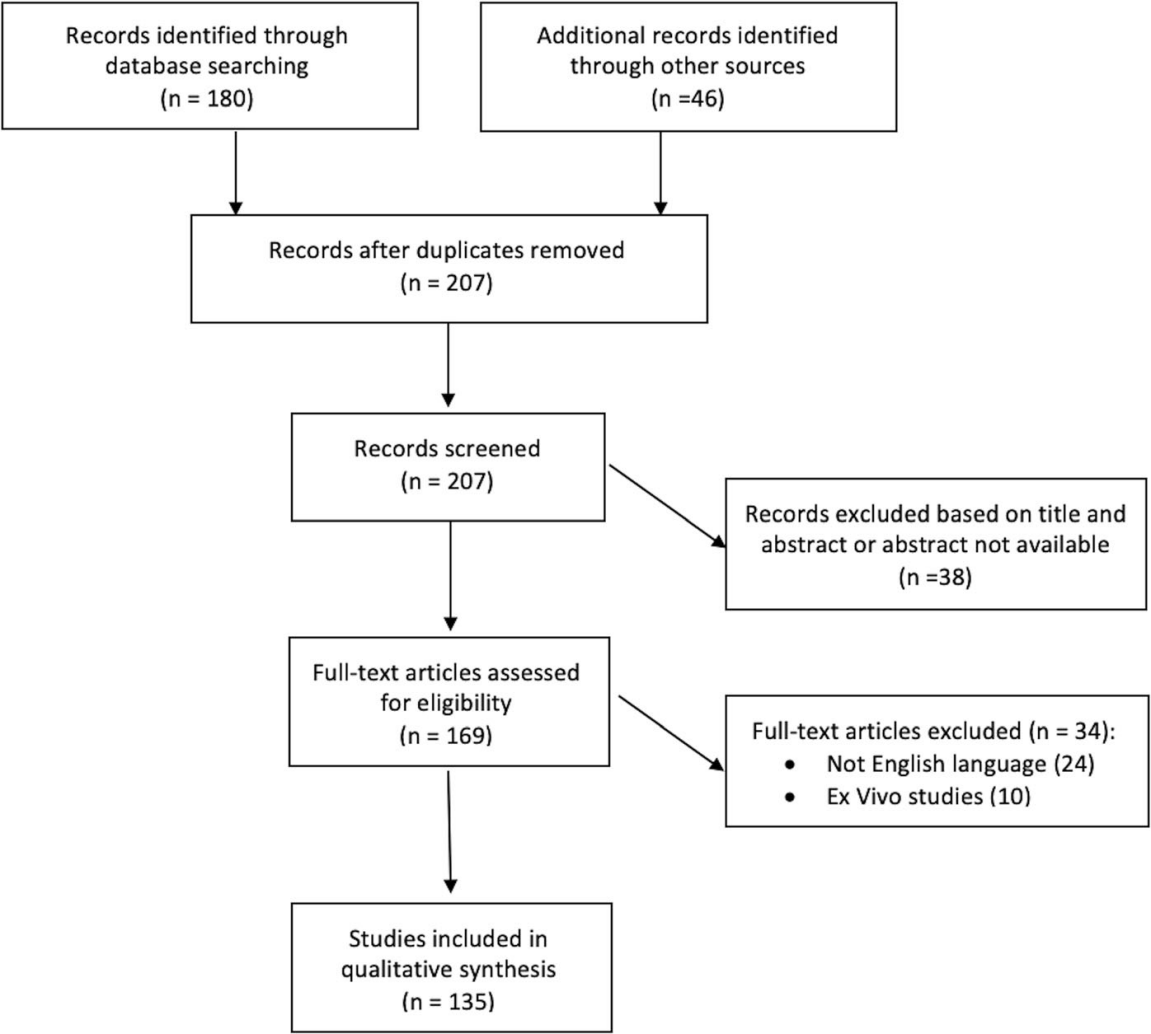
Review’s flow chart

A comprehensive search of the PubMed database was performed on 20 March 2020. The keywords used for the search were “ocular siderosis” and “siderosis bulbi”. The process applied for this review consisted of a systematic search of all available articles regarding OS. All identified electronic data captured were independently evaluated in terms of their titles and abstracts by two reviewers (M.M. and F.S.) to determine relevant articles. Additionally, the references of identified articles were manually checked to find any potential pertinent studies for review purposes. All studies available in the literature reporting original data on OS were initially included without restriction for study design, sample size and intervention performed. Articles written in languages other than English and ex vivo studies were excluded from the present review (Fig. 1).

## Epidemiology

OS may develop from 18 days to years after a penetrating ocular trauma [7, 8]. In particular, Kannan et al. described an OS that developed 12 years after injury [7]. Additionally, a case of OS development despite IOFB removal has been reported [9]. However, it resolved spontaneously successively, perhaps due to delayed IOFB removal or a second small IOFB that was initially undetected [9].

Recently, studies reported an average patient age of 22–25 years (range 18–67) with a vast majority being males [8, 10, 11].

Trauma occurred as a result of hammering in 41.67%, chiselling in 16.67%, lathe turning in 16.67%, electric welding in 4.17%, nail gun in 4.17% and unknown causes in 16.67% of cases [8]. Trauma occurred at work (54–72%) or at home (30%) [10]. Delayed presentation by patients (54.17%), missed diagnosis or delayed referral (25.00%), no history of trauma (16.67%) and undetected IOFB by computed tomography (CT) (4.17%) prolonged the interval from ocular trauma to IOFB discovery, which led to OS [8]. IOFB injuries are less common in people wearing eye protection; therefore, the usage of protective glasses should be recommended [12, 13].

IOFBs can be detected in 18–41% of penetrating globe injuries, and 78–86% are metallic [6]. Iron is the most common component of metallic IOFBs, followed by lead [6]. In 60–80% of cases, the entry wound is located at the cornea or corneoscleral junction, but several times it is not possible to detect it [11, 14]. IOFBs are localized in the posterior segment in 58–88% of trauma, in particular, 75% intravitreal, 19% intraretinal and 6% subretinal, typically in the inferior quadrants [11, 15]. Remarkably, IOFB location may be due to gravity and a sudden velocity reduction when it enters the vitreous cavity [2].

To date, misdiagnosed IOFB is responsible for up to 56% of malpractice suits related to ocular trauma [16].

It is important to note that all cases of metallic IOFB do not result in OS. Hwang et al. reported an iron IOFB embedded in the optic nerve of a 33-year-old man, suggesting that iron diffusion through the optic nerve axons may not occur [17]. In contrast, an embedded iron IOFB in the sclera or the lens may cause OS because iron may leak out and diffuse, especially if the IOFB is subcapsular or into the cortex [18–20].

Finally, OS is unlikely to occur in systemic iron-overload disease due to the blood–retina barrier [21].

## Pathogenesis

OS is caused by the interaction between trivalent iron ions and proteins; in particular, tissue alterations close to an IOFB are called “direct siderosis”, while those far from the IOFB are named “indirect siderosis” [22, 23]. The former is caused by hydroxyl radical formation due to a chemical reaction (Fenton’s reaction) that occurs when iron-binding proteins, such as apoferritin, are saturated, and an excess of free iron interferes with cellular enzymatic activity, causing lysosome breakdown [24, 25]. Consequently, pyknosis and degeneration of photoreceptors and RPE occur, along with inner retinal oedema. Nevertheless, the latter resolves in a few days [26]. Notably, rods are more susceptible to iron toxicity than cones due to different protective systems against oxidative and retinopathic factors, such as vitamin A deficiency and light-induced degeneration [26].

Moreover, iron ions could spread from the IOFB to photoreceptors through Müller cells, as Tawara et al. reported; in addition, RPE cells can be damaged through an indirect route [25]. In particular, ions migrate across the vitreous in a posterior–anterior way to the aqueous. Then, they penetrate the deep cornea layers and trabecular meshwork, which allows them to reach the suprachoroidal space and diffuse to the choroid and Bruch’s membrane until reaching the retinal pigment epithelium (RPE) posteriorly [27, 28].

In summary, in the early phase, OS histologically shows damage to photoreceptors and RPE cells sparing Bruch’s membrane and the choroid. In addition, inner retinal layers are not hurt, despite their proximity to the IOFB [26].

In contrast, indirect siderosis is characterized by damage to the retinal vessel named “vascular siderosis” [5]. In particular, iron has an affinity for acid mucopolysaccharides, one of the main components of vascular adventitia that cause toxic microvasculopathy and uncontrolled macrophagic activity. This phenomenon is responsible for the consequent degeneration of internal retinal layers, supplied only by retinal capillaries [5, 29]. These alterations add to photoreceptors and RPE changes already present in the early phase. Therefore, in chronic OS, all retinal layers are involved [30].

In summary, three phases characterize OS development. First, a latent period following injury without clinical signs manifests, which is variable from a few weeks to some years. Then, iron spreads within intraocular tissues with a high affinity for epithelial cells. Finally, tissue degeneration occurs, particularly in the retina and RPE [1, 31].

OS development and severity depend on several factors, such as IOFB dimension, shape and composition; IOFB localization; and associated trauma tissue reaction [32]. Regarding the shape, an irregular roughened IOFB develops OS quicker than a smooth and regular IOFB [1]. Moreover, small IOFBs may completely oxidize, so OS regresses [1]. Additionally, the IOFB iron content plays an essential role in OS development; the higher it is, the quicker the OS develops [1]. Regarding IOFB location, if it is in the vitreous or aqueous, OS occurs more rapidly than if it is encapsulated in tissues with low metabolism, such as the lens and the cornea [1].

## Diagnosis

When OS develops, the patient may experience nyctalopia and decreased colour vision [1]. Gradual vision impairment and progressive visual field loss are late symptoms [1]. In particular, young patients may develop consequential strabismus or amblyopia; therefore, follow-up is mandatory [33].

Anterior segment prevalent OS features are cataract development, iris heterochromia, pupillary mydriasis and secondary open-angle glaucoma [2, 31].

Usually, iron stains corneal epithelial cells during injury; however, iron granules may be deposited in any layer of the cornea, with a preference for deeper layers [34, 35].

Heterochromia is the earliest OS sign, especially in light-coloured irises [34]. It is due to iron deposition in the iris stroma and epithelium, and sometimes it is responsible for the patient seeking medical advice [34, 36].

The pupil can be dilated and nonreactive or with light/near reaction dissociation [37]. This phenomenon is named “iron mydriasis”, and it is a parasympathetic neuropathy due to iron precipitation in the sphincter and iris dilator muscles. Iris may show a hypersensitive response to diluted pilocarpine [37]. Thus, OS should be considered in the differential diagnosis of tonic or Adie’s pupil, which is caused by alteration of sphincter pupillae and ciliary muscle postganglionic parasympathetic innervation, usually after a viral illness [2, 38].

Moreover, brownish or rusty spots can develop on the anterior lens capsule due to iron deposition in epithelial cells, and sometimes, the lens itself could appear yellowish with cataract development [39]. The last phenomenon can also be due to penetrating trauma [40, 41].

Glaucoma is due either to iron deposits in the trabecular meshwork or to the production of albuminous aqueous by the ciliary body, which increases the intraocular pressure. Finally, trabecular fibrosclerosis can develop [1, 7]. Therefore, glaucoma in OS is medically uncontrollable and may require drainage surgery or eye enucleation [8, 42].

**Fig. 2:**
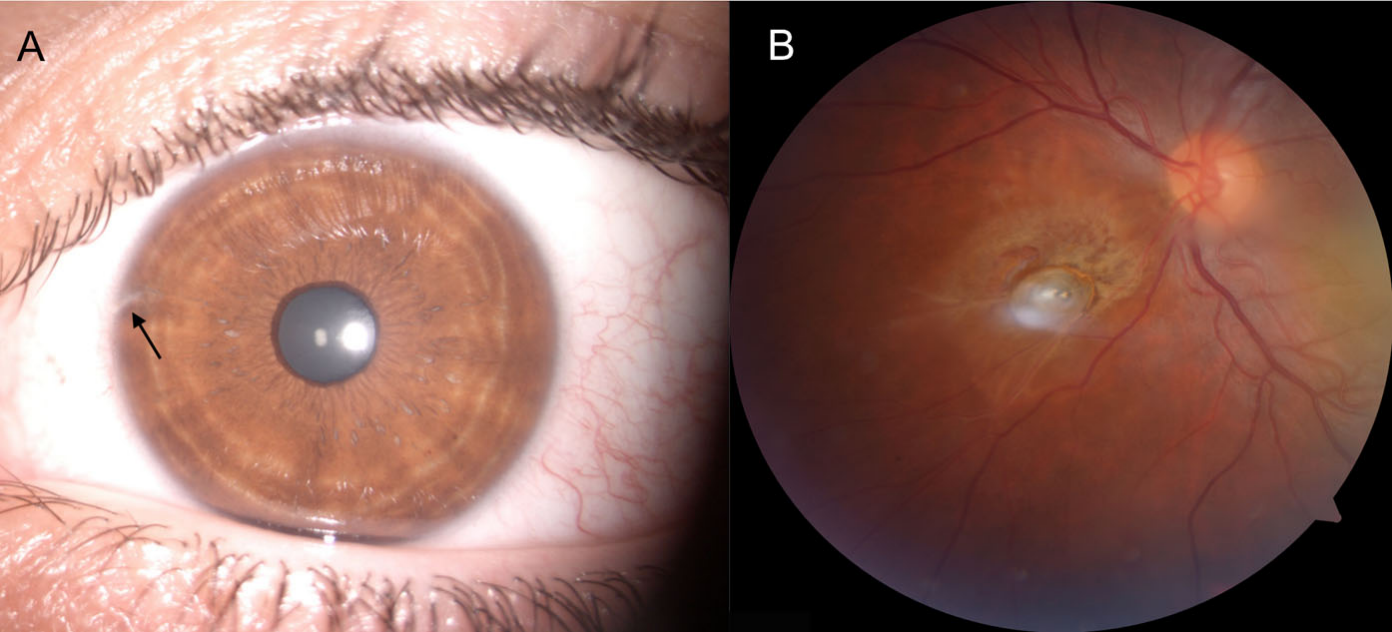
Patient with a history of high-velocity metallic injury. **a** No clinical signs of OS are detectable; however, a leukoma at the limbus attests to the IOFB entrance (arrow). **b** At the fundus examination, a metallic foreign body located nasally to the optic nerve head

Unusually, OS can manifest as uveitis, with the involvement of the anterior or posterior segment, or as panuveitis [43, 44]. Posterior uveitis does not occur unless Bruch’s membrane is damaged [1] (Fig. 2).

Regarding the posterior segment, OS causes retinal arteriolar narrowing and sheathing with pigmentary retinal degeneration similar to retinitis pigmentosa [45, 46]. Optic neuropathy with optic disc swelling or hyperaemia and cystoid macular oedema can also be observed [2, 47–49].

To avoid OS, in the case of a penetrating ocular injury, an IOFB must be excluded, especially after a history of high-velocity metallic injury, the obtainment of a detailed history and performance of a complete ophthalmic evaluation [42]. Therefore, it is mandatory to ask when and how the injury occurred [42]. Questions about pre-existing ocular conditions such as amblyopia or retinal diseases may be helpful [11]. Ophthalmic evaluation should include VA and pupil reaction assessment, extent and location of the wound, and the presence of hypopyon or other signs of endophthalmitis [11]. Then, if the cornea, lens and vitreous are clear enough, an accurate examination of the posterior segment should be performed to look for any retinal tears and to localize the IOFB [11].

The IOFB location depends on several factors. Sharp-edged fragments usually have a high speed and enter the anterior segment with less energy dissipation so they can more easily reach the posterior segment [42]. These IOFBs are usually small metallic fragments, called knife-edges, generated by pounding metal against metal during activities such as hammering or high-speed grinding [50]. In contrast, blunt objects require a great deal of momentum to penetrate because they have greater concussive force on the eye [50]. Usually, they cause generalized damage to the intraocular structure, even without ocular penetration [11, 14].

Entrance wounds should be identified if possible, perhaps using a Seidel test [42]. Usually, an anterior wound entrance is visible or indirect signs are noted, such as haemorrhage over the sclera, corneal oedema or a hole in the iris. The entry wound and the iris hole provide information about the IOFB trajectory [51]. Moreover, the wound length foretells the risk of retinal damage [14]. In particular, a shorter wound means less energy dissipation during penetration, so the IOFB may travel much farther inside the eye, reaching and injuring the retina [14]. Additionally, wound location has a predictive role because IOFBs entering through the sclera are more likely to cause significant damage than those entering through the cornea [42]. The cornea has an increased resistance compared to the sclera because it is constituted by parallel collagen fibrils bound together with mucopolysaccharides, which are perpendicular to the one above and below [50].

Finally, it is important to remark that intraretinal IOFBs are associated with an increased incidence of epiretinal membrane development, ranging from 29 to 36% of cases, and proliferative vitreoretinopathy (PVR) in 50% of cases, which develop retinal detachment in 79% of cases [52, 53].

## Imaging

Multiple imaging modalities, such as orbital computed tomography (CT), magnetic resonance imaging (MRI), ultrasonography and optical coherence tomography (OCT), help in IOFB detection when it has not been found during ophthalmological evaluation. Instead, electroretinography (ERG) is fundamental to assess retinal damage due to OS, but fluorescein angiography (FA), electrooculogram (EOG) and visual field provide valuable information.

### Orbital CT scan

**Fig. 3:**
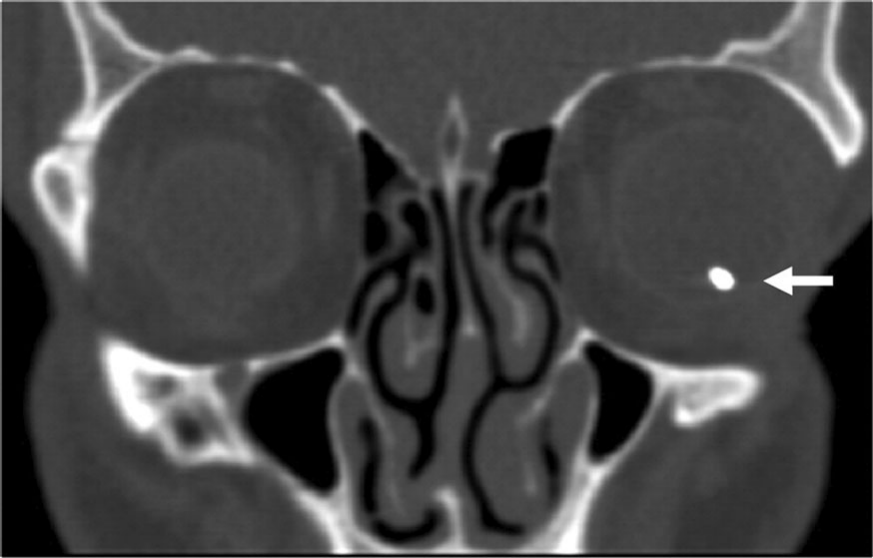
Patient with ferrous IOFB. CT coronal-section image shows a 4-mm metallic IOFB (arrow) inside the left globe. Reproduced with the permission from Pinto et al. (2012). Role of computed tomography in the assessment of intraorbital foreign bodies. Semin Ultrasound CT MR 33 (5):392–395. 10.1053/j.sult.2012.06.004 under CC-BY-NC-ND license

Orbital CT without contrast is the gold standard to identify metallic IOFBs, as it provides information about its localization (intraocular, extraocular or retroocular) and its size, with a sensitivity of 45–65% for IOFBs < 0.06 mm^3^ and of 100% for IOFBs > 0.06 mm^3^ [54, 55]. Sagittal and coronal thin (1.0–1.5 mm) scans through the orbit are warranted [56]. Two types of CT imaging are available: conventional CT and helical/spiral CT [57]. The former is more widespread than the latter, but helical CT is quicker, reducing radiation exposure and allowing a better multiplanar reconstruction with fewer motion artefacts [57]. Nevertheless, patient movements are still the most common cause of undetected metallic IOFBs during CT acquisition [14, 58]. Finally, orbital CT is highly sensitive, requires little patient cooperation and avoids global manipulation [58–61] (Fig. 3).

### Magnetic resonance imaging (MRI)

Compared to CT, MRI is superior in detecting and identifying nonmagnetic IOFB, with a sensitivity of 95%, and offers a better definition of orbital soft tissue without using ionizing radiation [62, 63]. However, it is not widely accessible, it is time-consuming, and it is contraindicated if metallic IOFB is suspected [64, 65]. During MRI, metallic IOFB movement is rare; nevertheless, it can cause further ocular damage [66–68]. Therefore, MRI should be reserved only for patients with suspected ocular injuries not detected by CT, such as subtle open-globe injury due to the non-metallic foreign body [63].

### Ultrasonography

B-scan ultrasound (brightness modulation) was first used by Okasala and Lehtinen in 1957 for IOFB detection [69]. It generates a picture displaying top to bottom and proximal to distal sound reflection from the transducer. B-scan is a dynamic examination providing real-time, two-dimensional images of the ocular and adjacent tissues with a high resolution (0.1–0.01 mm) [70]. It can detect both metallic and non-metallic IOFBs. It is sensitive and specific in the case of media opacity such as hyphema or corneal opacity; however, it requires an expert operator for differentiating artefacts from the IOFB itself [71].

**Fig. 4:**
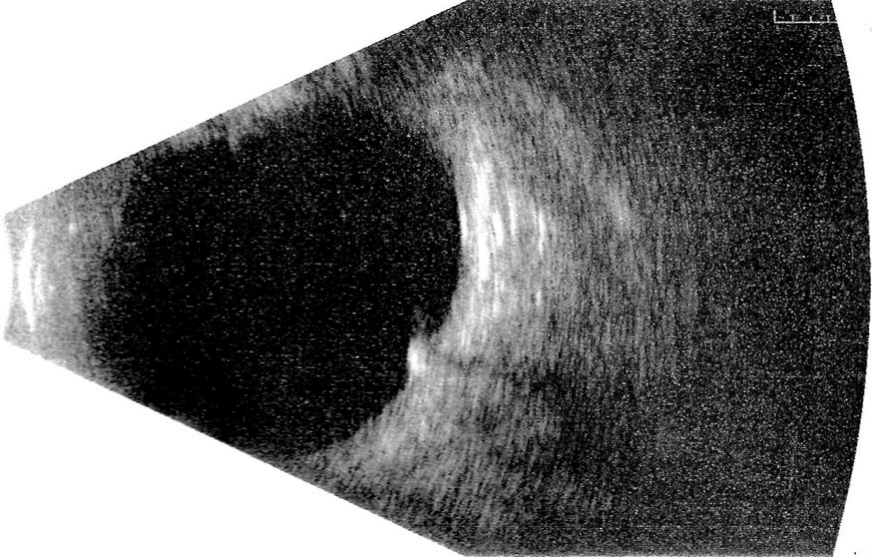
Patient with a ferrous IOFB. B-scan detects the IOFB as an echo-dense signal with comet trail artefact. It also rules out vitreous haemorrhage, retinal detachment and PVR

IOFBs appear as an echo-dense signal with posterior shadowing (multiple small hyperechogenic bands), named the “comet tail artefact”, due to metal that generates multiple internal reflections [71–73]. Nevertheless, the B-scan tends to overestimate IOFB size, so it should not be used for measuring purposes [70, 74]. However, it is useful in identifying associated tissue injuries such as choroidal and vitreous haemorrhages or retinal detachment [58, 75, 76] (Fig. 4).

Ultrasound biomicroscopy (UBM) uses high-frequency (50 MHz) sound waves with a higher resolution, reaching a maximum depth of 5 mm [77]. Compared to CT and B-scan, UBM has an increased sensitivity to identify IOFBs located in the anterior chamber [78]. It can detect anteriorly located IOFBs, for example, in the angle or near the ciliary body, ciliary processes and retrolental space [77].

However, both B-scan and UBM should be avoided in open-globe injury, and if suspected, the operator should be prudent [79].

### Optical coherence tomography (OCT)

**Fig. 5:**
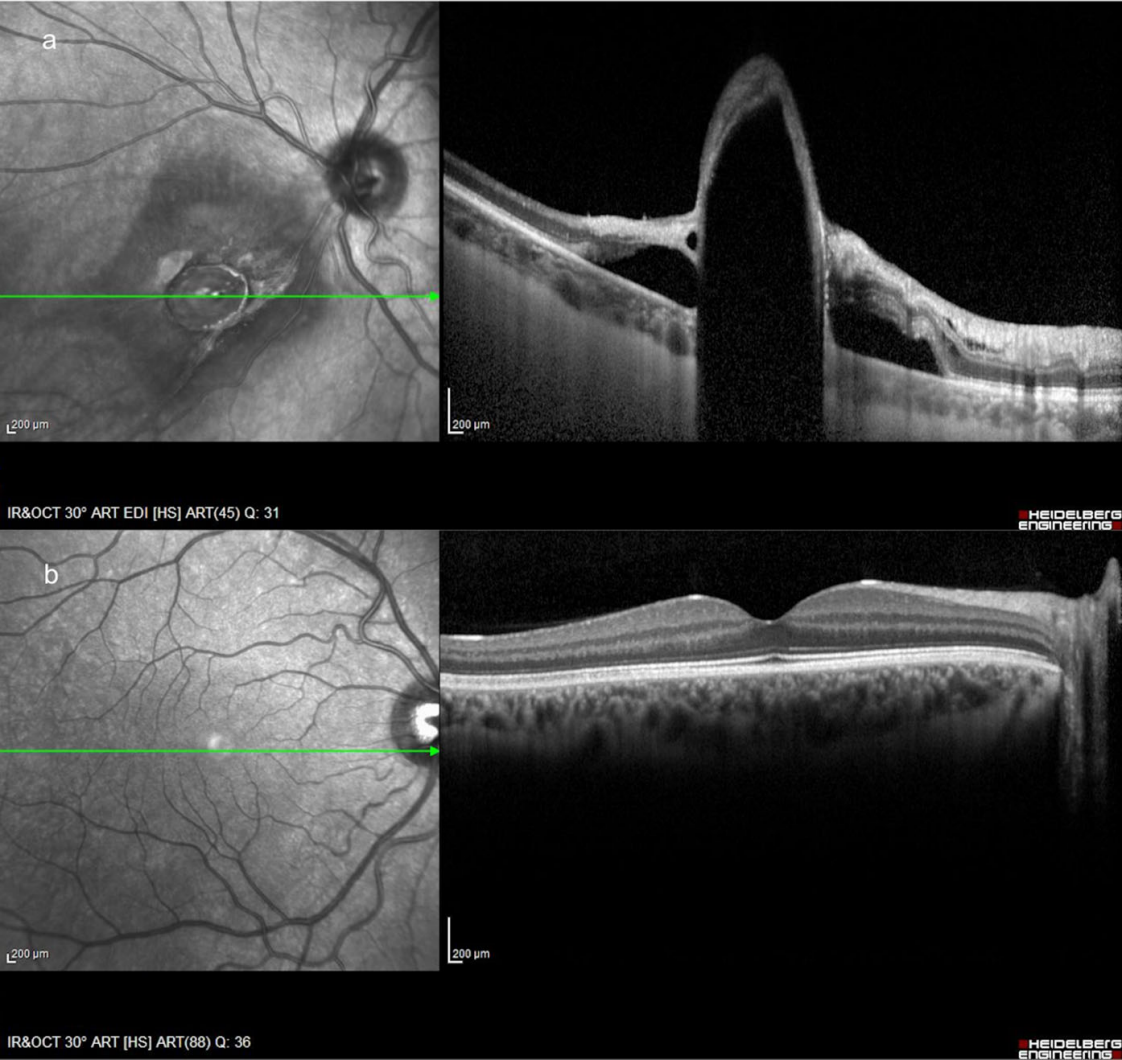
Patient with a ferrous IOFB. **a** OCT shows that the IOFB is covered by a fibrous membrane, which encapsulates it. **b** Moreover, OCT excludes any epiretinal membrane or oedema at the macula

In OS, OCT was used first by Chao et al. to identify the encapsulation of an IOFB [80]. In particular, OCT can analyse the relationship of an IOFB with adjacent tissues for pre-operative planning, e.g. highlighting if the IOFB is subretinal, intraretinal or epiretinal when it is located in the posterior segment [80]. OCT also shows whether an IOFB is embedded or not by a fibrous membrane, helping surgeons in the decision-making process on whether the removal is urgent or not because IOFB encapsulation slows OS development [1, 80]. Moreover, OCT detects macular pathologies, such as epiretinal membrane or oedema, which may be hardly identified by fundus examination [29, 80] (Fig. 5).

### Electroretinography (ERG)

ERG is the gold standard to detect and follow-up retinal toxicity secondary to OS, often displaying rod-cone functional anomalies [81–84]. Different typologies of ERG are available: focal ERG (fERG), full-field ERG (ffERG), pattern ERG (PERG) and multifocal ERG (mfERG). fERG measures the functional integrity of the fovea and can be useful in cases of age-related macular degeneration [85]. In contrast, ffERG measures the stimulation of the entire retina with a full-field flash stimulus under dark-adapted (scotopic) and light-adapted (photopic) conditions. It is useful for detecting generalized retinal dysfunction, such as toxic retinopathies and cone–rod dysfunction; however, it cannot identify small areas of abnormality [86, 87]. PERG provides information about optic nerve integrity stimulating macular and retinal ganglion cell function with a contrast-reversing pattern, usually a black and white checkerboard [88]. Finally, mfERG produces a topographical map of retinal function, scaling the stimulus according to photoreceptor density across the retina, with a lower density at the fovea and a higher density at the periphery [89]. In particular, mfERG waves are similar to those recorded by the ffERG with an initial negative deflection, called N1, followed by a positive deflection (P1) and a second negative deflection (N2) [90]. In mfERG, both waves are evaluated on five rings with ring 1 representing a < 2° field, ring 2 representing a 2°–5° field, ring 3 representing a 5°–10° field, ring 4 representing a 10°–15° field and ring 5 representing a > 15° field. All these measurements are summarized in a three-dimensional plot [91–93].

**Fig. 6:**
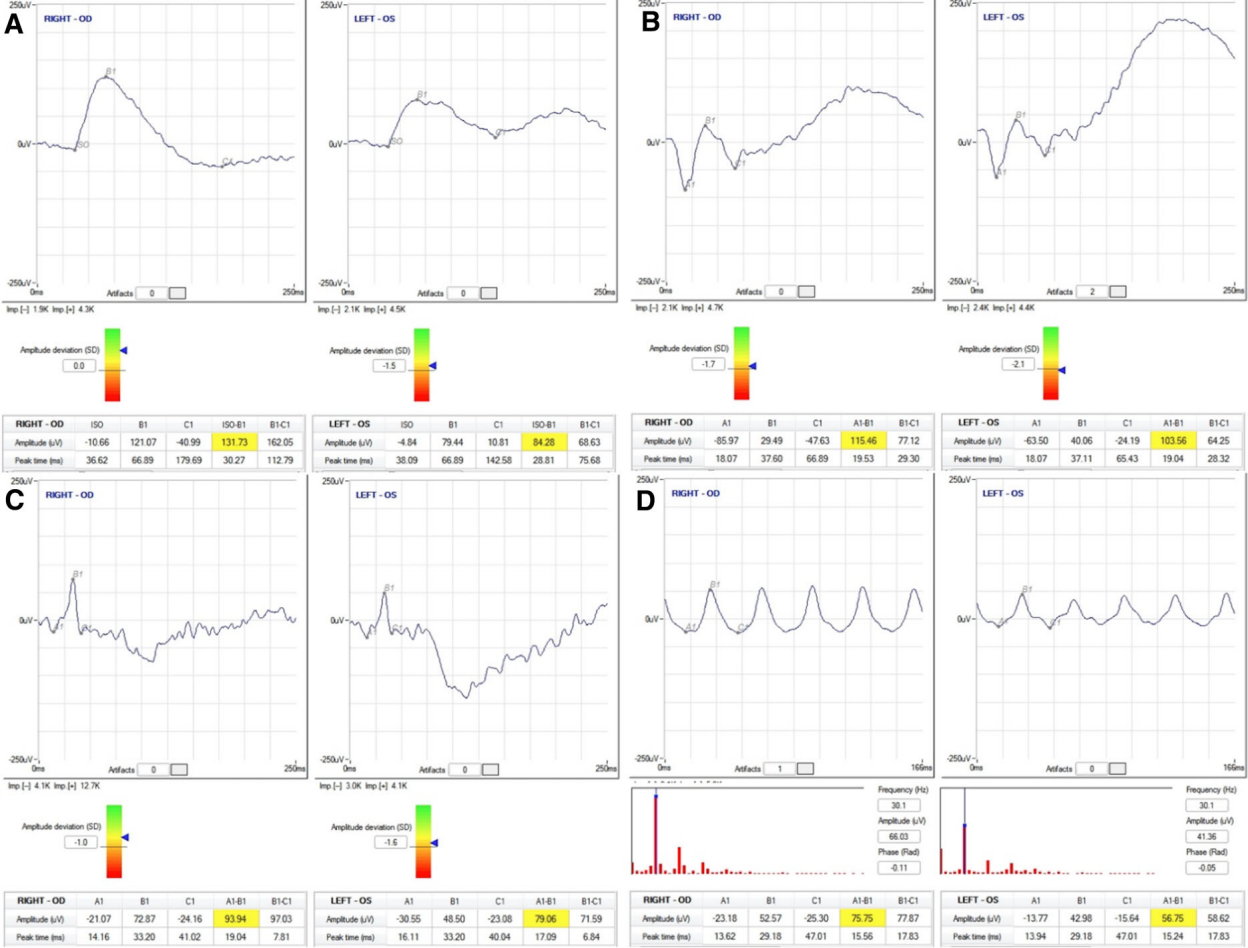
Patient affected by OS. ERGs recorded according to ISCEV standards. Electrode type: HK-LOOP; recording system RETIMAX ADVANCED (CSO, Florence, Italy). **a** Scotopic ERG-dark adapted 0.01 Flash strength 0.010 cd s m^−2^, frequency 0.5 Hz, 20 min of dark adaptation. Normal values according to age ≥ 72 μV. Left eye (LE) *b*-wave amplitude is reduced by approximately 36% compared to the right eye (RE) (RE 131.73 μV vs. LE 84.28 μV). **b** Scotopic ERG-dark adapted 3.0 Flash strength 3 cd s m^−2^. Frequency 0.1 Hz. Normal values according to the age ≥ 127 μV. The LE *b*-wave amplitude is slightly reduced. **c** Photopic ERG-light adapted 3.0 Flash strength 3 cd s m^−2^, frequency 1 Hz, background luminance 30 cd m^−2^, 10 min of light adaptation. Normal values according to the age ≥ 69 μV. Both amplitudes are within the normal value; however, the LE *b*-wave is slightly reduced compared to the right eye. **d** Flicker ERG-light adapted 3.0 Flash strength 3 cd s m^−2^, frequency 30 Hz, background luminance 30 cd m^−2^, 10 min of light adaptation. Normal values according to the age ≥ 30 μV. The LE *b*-wave amplitude is reduced by 25% compared to the RE (RE 75.75 μV vs. LE 56.75 μV), and the peak times are similar

ffERG can be considered the gold standard for assessing retinal damage by iron IOFB, as it can detect functional abnormalities of the retina before any pathological changes are visible by fundus examination or fluorescein angiography [91, 94]. In the OS early phase, ffERG shows an increased *a*-wave (hypernormal) or an increased implicit time, indicative of subtle retinal toxicity [90]. After that, the *b*-wave decreases with a reduction in the *b*-wave/*a*-wave ratio (*b*/*a* ratio < 1). Finally, the amplitudes of the *a*-wave and *b*-wave progressively decrease to become undetectable [7, 95] (Fig. 6).

Knave classified ffERG changes during OS development [96]:

- type 1 or subnormal: *b*-wave amplitude is reduced to less than 75% of that of the unaffected eye and *a*-wave is abolished or greatly diminished
- type 2 or negative with two different subtypes:
  - the plus one (+) where the *b*-wave amplitude is greater than 75% of that of the unaffected eye and *a*-wave is increased,
  - the minus one (−) where the *b*-wave amplitude is less than 75% of that of the unaffected eye and *a*-wave is preserved
- type 3 or extinguished: flat response
- type 4 or supernormal: *b*-wave amplitude is greater than 125% of that of the unaffected eye and *a*-wave amplitude is not diminished.

The pathogenesis of type 4 *b*-waves is unclear. This may be due to an increased level of cyclic guanosine monophosphate (cGMP) in the rod photoreceptors or to a change in the extracellular potassium concentration, causing an abnormal photoreceptor response [97, 98]. This finding may represent the earliest ERG alteration in OS; however, it can also be detected in other ocular diseases (e.g. albinism, hyperthyroidism, optic atrophy, corticosteroid administration and retinal vascular disorders) [32].

Another early finding in OS is oscillatory potential amplitude reduction, which records the activity of inhibitory synaptic circuits within the inner plexiform layer. This alteration represents an irreversible change in the inner retina layers occurring when the amplitude of the *a* and *b* waves is still within the normal limit [94].

After IOFB removal, ERG amplitudes may increase, confirming that iron toxicity is reversible in the early period of the disease [94, 99]. In particular, Tanabe et al. reported that type 2 positive *b*-waves represent an early stage of OS and that removal of the IOFB at this time can restore a normal ERG record [32]. In the absence of free ferrous ion release, macrophages may detoxify and store the remnant free ions [91]. Otherwise, ffERG amplitudes may remain subnormal due to perivascular iron deposits. They cause toxic microvasculopathy and uncontrolled macrophagic activity with subsequent photoreceptor atrophy, as mentioned above [7, 21, 22, 81, 100].

Finally, ffERG is also useful for follow-up with patients who prefer to avoid surgery or with an IOFB that is challenging to remove [49].

**Fig. 7:**
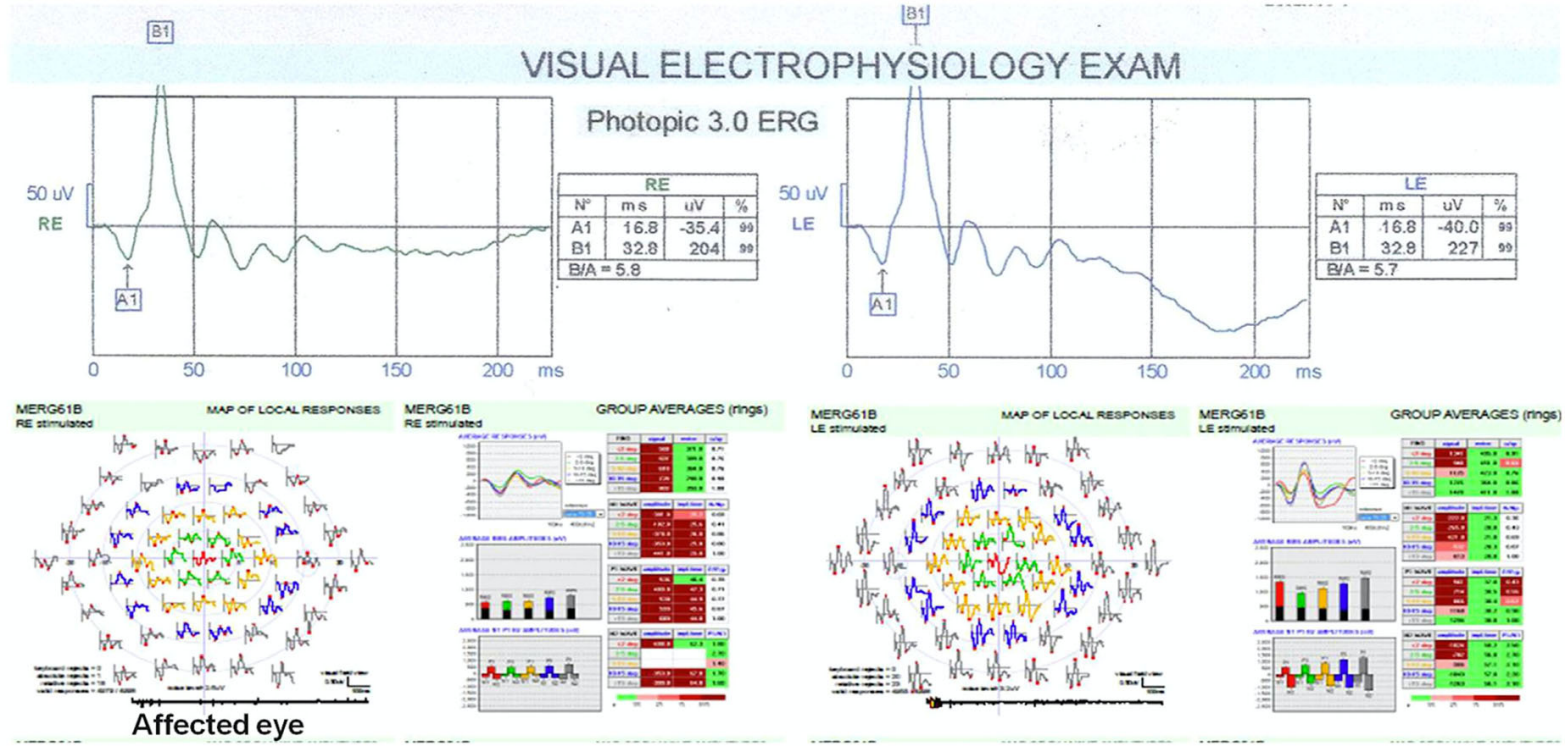
Patient with a ferrous IOFB. ffERG shows the average values of the amplitude and the latency, and the ratio of *b*/*a* is almost similar between the two eyes. Instead, mfERG of the same patient shows an abnormal implicit time for the 2° ring of the affected eye. The coloured three-dimensional plot also shows a remarkable depressed foveal peak for the P1 wave in the affected eye. Reproduced with permission from Ref. [90]

Recently, Sahay et al. reported that mfERG might reveal subtle electrophysiological retinal dysfunction in eyes affected by OS, even before ffERG [91]. In particular, P1 and N1 wave amplitudes were reduced in all five retinal rings, especially the inner rings, when ffERG showed no difference in either *a*- and *b*-wave amplitude or peak time. The greater sensitivity of mfERG may be due to the higher density of RPE and photoreceptors in the fovea [91]. The same authors reported that implicit time delay might be an early predictor of ferrous toxicity, and it is diagnostically superior to amplitude wave reduction [90]. Direct iron-induced cone dysfunction or an impaired cone–rod interaction could be the causative mechanisms [90]. Moreover, Sahay et al. reported an improvement in the P1 amplitude wave on mfERG after surgery. However, P1 did not recover entirely until six months postoperatively, and the N1 amplitude and the peak time of both the P1 and N1 waves remained altered [91]. The same authors suggested considering the peak time of the P1 wave as a permanent marker for previous retinal dysfunction due to OS [91] (Fig. 7).

### Fluorescein angiography (FA)

OS can cause multiple ocular alterations, especially in the posterior segment; therefore, FA can play an important role [101]. In particular, retinal capillary nonperfusion, retinal vessel narrowing and RPE changes can be observed, mimicking retinitis pigmentosa [30, 102]. Cystoid macular oedema can be displayed on FA with a petaloid pattern due to dye accumulation in microcysts in the outer plexiform layer [53]. Finally, FA can detect delayed macular ischaemia with perifoveal arcade staining and perivascular spots, presumed to be iron deposits. This phenomenon may cause VA reduction one year after IOFB removal [103].

### Electrooculogram (EOG)

EOG assesses the function of the RPE and photoreceptors by measuring changes in corneal–retinal standing potential (the difference between the retina and the electropositive cornea) during the dark-adapted and light-adapted states [104]. Nevertheless, diffuse RPE damage is needed to significantly affect the EOG response, limiting its role in OS diagnosis and follow-up [105].

### Visual field

**Fig. 8:**
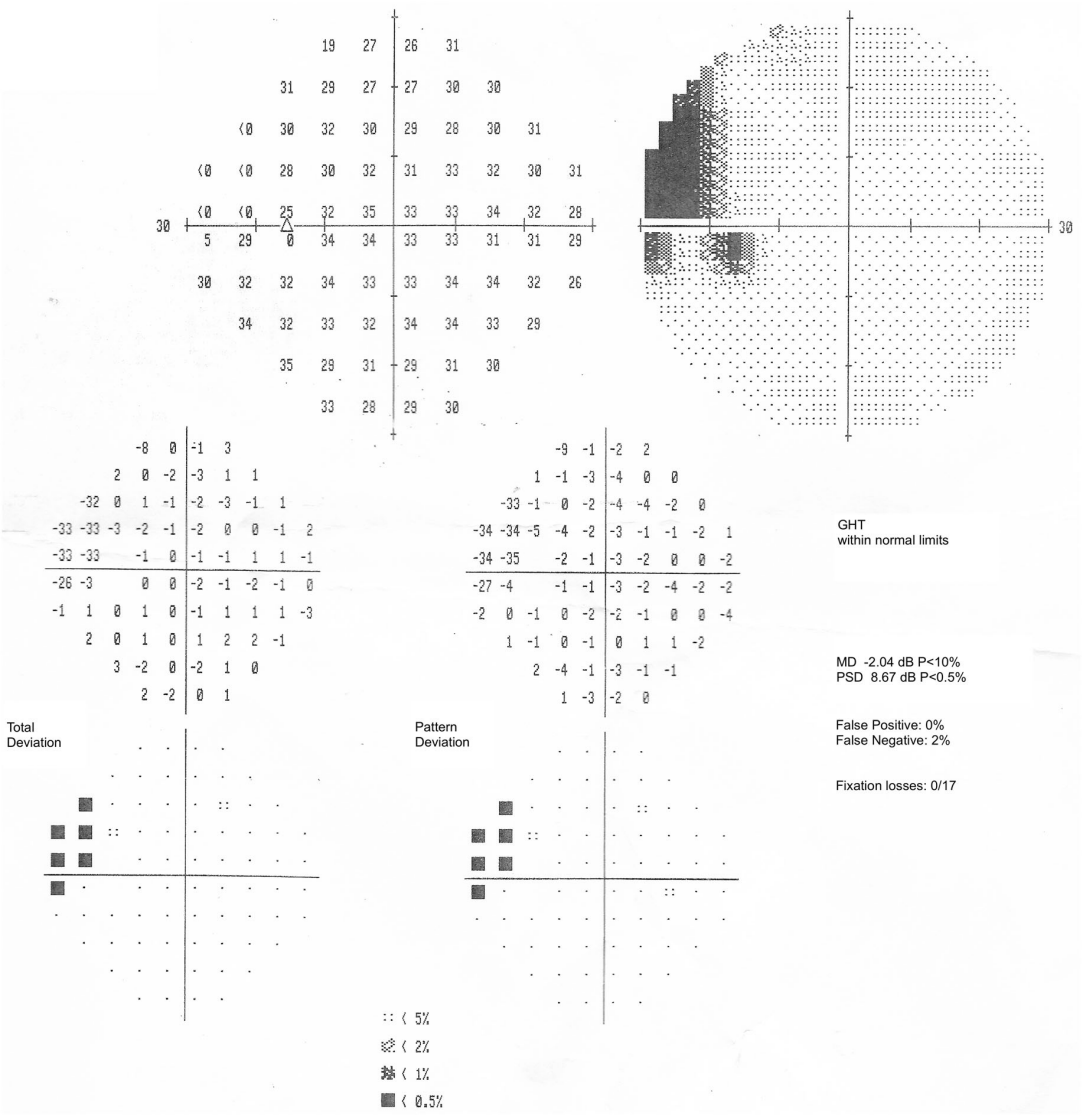
Patient with a ferrous IOFB in the left eye. The 30-2 visual field shows a temporal scotoma due to a peripapillary IOFB

In OS, progressive visual field narrowing has been reported; however, the exact cause is not known [80]. In advanced siderosis, retinal circulatory insufficiency could contribute to this phenomenon, but glaucoma development may also play a role [35, 106]. Moreover, if the IOFB is peripapillary or paramacular, it can cause a scotoma in the visual field [80] (Fig. 8).

## Treatment

### Fresh IOFB

The treatment of OS is an important motivation to remove all fresh ferrous IOFBs, in addition to an increased risk of infection. Therefore, it is essential to obtain cultures of aqueous and vitreous and to evaluate the patient’s tetanus status [107]. In particular, to prevent tetanus infection, vaccination and proper wound care are necessary [107]. Tetanus toxoid-containing vaccines protect all people for approximately ten years. Protection decreases over time, so adults need to obtain a Td (tetanus-diphtheria) or Tdap (tetanus-diphtheria-pertussis) booster shot every ten years [107]. If the patient is on schedule with vaccination, it is recommended to administer one boost of Td. However, if the last shot was performed more than ten years prior, it is mandatory to deliver one shot of Td and a shot of tetanus immunoglobulins [107].

Systemic and topical antibiotic therapy should be started as soon as possible against the most common pathogens, such as coagulase-negative Staphylococcus spp., Streptococcus spp., Bacillus spp. and Clostridium spp. [108–110]. In particular, a third- or fourth-generation fluoroquinolone–moxifloxacin 400 mg or levofloxacin 500 mg is advised to prevent post-traumatic endophthalmitis, administered once a day from the trauma to 7–10 days after surgery [107]. However, the best prophylactic regimen has not been determined [14, 109, 111].

The decision to perform prompt (within 12 h) or delayed (within 14 days) IOFB removal depends on its location [42]. In particular, if it is localized in the anterior chamber, the IOFB should be removed immediately [112, 113]. If it is into the lens, it can be removed during cataract surgery because cataract does not necessarily develop after a trauma, bearing in mind that iron particles may leak out, especially if the IOFB is at the lens periphery [112, 113].

Finally, if the IOFB is in the posterior segment, a careful analysis of the risks and benefits should be performed. The presence of PVR, vitreous haemorrhage or retinal detachment requires PPV [95]. In the case of an IOFB that is mobile in the vitreous cavity or a non-encapsulated IOFB in the retina, prompt removal is advisable [95].

Moreover, immediate surgery decreases the rate of endophthalmitis development, usually allowing a single surgical procedure, which is associated with a better visual outcome compared to multiple surgical procedures [114]. Nevertheless, Colyer et al. reported that delayed IOFB removal is not a significant risk factor in the development of post-traumatic endophthalmitis [115]. In a cohort of 70 patients, with an average period between trauma and IOFB removal of 38 days (range from 2 to 661 days), no OS, sympathetic ophthalmia or endophthalmitis was reported. No infection may be due to IOFB self-sterilization due to friction-generated heat during penetration, prompt broad-spectrum antibiotic administration and immediate globe closure [115].

Moreover, VA improvement is similar if the IOFB is removed immediately or within two weeks [95, 108]. In the latter case, corneal oedema may decrease without precluding posterior segment visualization, and posterior vitreous detachment develops, allowing a more straightforward posterior hyaloid excision during PPV [108]. However, it is important to remark that surgery cannot be delayed for more than 14 days. Over this period, the risk of PVR and tractional retinal detachment is too high [116].

### Chronic IOFB

In the case of chronic IOFB, it is fundamental to discuss all treatment options with the patient because IOFB removal may improve VA, signs of OS and ERG alteration, even if the *b*-wave amplitude is markedly reduced [95, 99].

IOFB removal is ruled by OS manifestation and IOFB location [42]. If OS is already developed, the IOFB must be removed [42]. Otherwise, if ERG does not show any sign of OS, surgery can be delayed if the IOFB is subretinal or inside a clear lens [42]. In the former case, OS development is usually low, so surgical risk outweighs the benefit, although in the latter case, the IOFB can be removed when cataracts develop [42]. Nevertheless, ferrous IOFBs located in the lens may still cause OS because iron ions leak out, especially if the IOFB is in the lens cortex or subcapsular [19]. Additionally, the fibrous tissue around the IOFB does not prevent OS, and any further intraocular manipulation may dislodge the IOFB, so once eye conditions are stable, they may not necessarily remain so [45, 95].

If the patient prefers to avoid surgery, regular follow-up is mandatory, with VA assessment, ophthalmoscopic evaluation, orbital CT or B-scan and ffERG [42]. ffERG is crucial because electrophysiological signs arise earlier than clinical signs [117]. However, if the patient is not expected to return for follow-up, IOFB removal should be recommended [42].

### Deferoxamine

Historically, medical therapy for systemic siderosis consisted of galvanic deactivation or intravenous EDTA or adenosine triphosphate [118]. In 1960, Bickel et al. developed deferoxamine from Actinomyces spp. [119]. Deferoxamine is a chelator agent with high iron affinity, and it is used in siderosis prevention due to iron overload in patients with thalassaemia subjected to multiple transfusions [120]. Wise et al. reported that deferoxamine subconjunctival injection (10–100 mg) was able to prevent the development of OS but not to resolve established cases because chelation can only remove free iron ions before becoming stored within cells [119]. Additionally, topical deferoxamine drops (concentration 10%) showed no clinical improvement [121]. It is crucial to remark that deferoxamine-related toxicity exists, both systemic (bone dysplasia and high-frequency sensorineural hearing impairment) and topic (RPE alteration, reduction in ERG amplitudes, nyctalopia and colour vision alteration, principally in the tritan axis) [122]. Therefore, deferoxamine use is not recommended in OS management [122].

### Surgery techniques

Metallic IOFB removal may be performed via an external approach [sclerotomy with an external electromagnet (EEM)] or via an internal approach associated with PPV if the IOFB is in the posterior segment [7, 29, 123].

Traditionally, Lancaster’s criteria ruled EEM applications: “Unless a giant magnet can pull a small steel ball 1 mm in diameter with a force of over 50 times its weight at a distance of 20 mm, and unless a hand magnet pulls such a ball in contact with its tip with a force over 5000 times its weight, they are not ophthalmologically effective” [42]. Consequently, the EEM tip design tried to increase the pull force and the distance at which this force is efficient [42]. In 1967, Bronson developed a handheld EEM with two magnet tips: one similar to the end of an acorn and the other similar to a lance. Removing IOFBs located in the posterior segment with EEM requires a sclerotomy and does not necessitate performing a PPV [42]. In particular, after a conjunctival peritomy, a sclerotomy of 1.50–3.00 mm at 4.5 mm from the limbus is performed. Then, the IOFB is extracted using an EEM and an indirect ophthalmoscope. Finally, the sclera is sutured, and cryopexy is performed [124]. Nevertheless, the alignment of the magnetic tip, the surgical incision and the IOFB are very challenging, and IOFB removal is not under the surgeon’s visual control [42]. Thus, IOFBs may lacerate the retina or anterior ocular structures such as the lens, zonules or ciliary body when it is rapidly pulled towards the tip of the magnet. In particular, IOFB removal with EEM is associated with a 23% risk of developing vitreous haemorrhage and 10% risk of developing endophthalmitis [124, 125]. EEM also tends to overheat and eventually burn the patient’s skin [126, 127]. Therefore, today, EEM can be used only to remove IOFB remnants to as low as 59% but is contraindicated to remove the whole IOFB [124].

The internal approach for removing IOFBs is performed using forceps or intraocular magnets (IOMs). Several surgical forceps are available: engulfing the IOFB or with serrated jaws to capture irregularly shaped IOFBs [128, 129]. Instead, IOM requires less surgical skill but can remove free-flying IOFBs less than 2 mm in diameter [42]. Nevertheless, concomitant forceps use is advisable because IOM loses its magnetic properties over time, as do ferrous IOFBs in the case of delayed removal [7].

If the IOFB is in the anterior chamber, the surgeon can perform paracentesis and try to dislodge the foreign body with balanced saline solution (BSS) or viscoelastic [42]. If it is not enough, the limbal incision can be enlarged to remove the IOFB with IOM or forceps [42]. It is important to note that if the IOFB is located at an angle, gonioscopy should be performed before and during surgery [56].

When the IOFB is in the ciliary body, its removal may require a scleral flap near the limbus or a trephine flap over the pars plana of the ciliary body [42]. In the case of irideal IOFBs, iridotomy or iridectomy could be necessary [14].

If the IOFB is in the lens, an accurate follow-up option is to delay removal until cataract develops [42]. However, iron particles may leak out if the IOFB is in the lens cortex [112]. Another option is to remove the IOFB from the lens without performing lensectomy; however, cataract development will be hastened [113].

If the IOFB is in the posterior segment, a 23-gauge PPV is usually performed [42]. Once the IOFB is localized, the vitreous surrounding it is removed, and the IOFB is released out if encapsulated [42]. Remember to perform a barrage laser treatment if a consequent retinal tear is suspected [42]. At this point, the IOFB removal is performed using forceps or IOM, and vitrectomy is completed. IOFB removal can be performed through the limbus or the sclera, depending on its diameter [42]. If it is larger than 6 mm, removal through the corneoscleral region is preferred; instead, if it is smaller, removal through the sclera can be performed, enlarging the sclerotomy with a T or L shape [130]. Consequently, the infusion pressure should be increased to maintain proper globe contour. Finally, the expanded sclerotomy site is closed using absorbable sutures and the conjunctive reapproximated [130].

Interestingly, Singh et al. reported a 23-gauge transconjunctival sutureless vitrectomy for removing metallic IOFBs [131]. Fourteen patients with IOFBs retained in the posterior segment after a penetrating trauma involving the cornea or the lens were enrolled. After PPV and lensectomy, a superior self-sealing limbal incision was performed to grasp and remove the IOFB with 20-gauge forceps [131].

A newer surgical technique, named the “handshake technique”, has been described by Dhoble et al. [128]. In particular, an IOFB is removed through a sclerocorneal tunnel using two IOMs introduced through PPV sclerotomies. This procedure is suitable for all sizes of IOFB without needing sclerotomy enlargement. The second IOM is fundamental to hold and deliver the IOFB safely outside. This technique is suitable for IOFBs with irregular shapes or diameters greater than 5 mm [128]. In a series of ten patients in which the abovementioned technique was applied, the final VA was 20/60 or better in seven of them, between 20/200 and 20/80 in one and counting fingers or less in two [128].

Recently, 25 gauge PPV has been described to remove metallic IOFBs [132, 133]. Nevertheless, Kiss et al. enlarged the original sclerotomy site with a 20-gauge MVR blade to the appropriate size, ensuring adequate space for IOFB removal [132]. Instead, Kurnikata et al. extracted the foreign body through a posterior capsulorhexis, an anterior continuous curvilinear capsulorhexis and the corneal incision (triple C-through technique) [133].

Mester et al. compared EEM versus PPV with IOM or forceps [127]. In 30 eyes, the IOFB was removed with EEM, while in the other 34 eyes, the IOFB was removed with PPV within 14 days of the injury. In the EEM group, 48% of cases reported PVR, 23% of cases vitreous haemorrhage and 10% of cases endophthalmitis. Visual acuity (VA) improved in 23% of eyes and decreased in 53% of cases. In the PPV group, PVR developed in 12%, and no endophthalmitis or other iatrogenic damage was reported. VA improved in 68% of cases and deteriorated in 15%. The mean postoperative VA in the PPV group was significantly higher than that in the EEM group (*p* = 0.001). Therefore, these authors concluded that PPV is superior compared to EEM [127].

When OS develops, the IOFB should be removed; as discussed before, however, there are few published studies on the clinical outcome [7, 8, 21, 32, 42, 99, 134].

Hope-Ross et al. described their experience in eight patients with OS [21]. Seven had a history of trauma; in three of them, the diagnosis of IOFB was missed even though they presented to an emergency department at the time of injury. OS was diagnosed between 2 and 24 months after the injury. The *b*-wave was subnormal in all patients and did not recover after IOFB removal. The IOFB was removed in seven cases (four with EEM and three with PPV and forceps). The final VA was light perception in two patients due to the development of PVR; however, in other cases, it was 6/12 or better. Follow-up ranged from 3 months to 84 months [21].

Kannan et al. treated nine eyes with OS [7]. The mean patient age was 31.6 years. Trauma occurred from 3 months to 12 years before OS diagnosis, with an average retained time of 2.9 years. Baseline VA ranged from 1/60 to 6/9, and the most common signs were cataract and pigmentary retinal degeneration. ERG was subnormal in all nine eyes, and the IOFB was removed using PPV in all cases. ERG improved in 78% of patients, and 78% of eyes gained two or more VA lines after surgery. Follow-up ranged from 6 months to 3 years. Complications encountered postoperatively were retinal detachment (1) and retinal tear (1) [7].

Kunh et al. reported a case of OS that developed two years after minor ocular trauma in a 37-year-old man [99]. He presented with iris heterochromia and occasional vision blurring. VA was 20/20, and the ocular exam revealed a sealed corneal perforation with a corresponding iris hole. The IOFB was located in the inferotemporal quadrant of the retina. ERG showed a *b*-wave reduction of 50%. The IOFB was removed with IOM during PPV. At three months of follow-up, VA was 20/20, and the *b*-wave almost completely recovered [99].

Bloom et al. described a case of a 20-year-old man with gradual visual loss and cataract development over six months [134]. VA at presentation was hand motion. The B-scan was inconclusive, so a CT scan was ordered, revealing the presence of an IOFB. Combined PPV with cataract extraction was performed to remove it. One month after surgery, VA improved to 20/20, although the ERG showed a *b*-wave amplitude reduction [134].

Recently, the ocular trauma score (OTS), which is a prognostic model to predict the visual outcome in ocular injuries, has been evaluated in OS [8, 135]. In particular, Zhu and colleagues enrolled 24 patients with OS who underwent surgery to remove IOFBs [8]. The most common OS signs were cataract (95.83%), retinal pigmentary degeneration (68.18%), iris heterochromia (58.33%) and pupillary mydriasis (47.62%). The IOFB was removed in 22 cases (91.67%), except for two enucleated eyes due to absolute glaucoma. VA improved in 63.64%, remained stable in 18.18% and was reduced in 18.18% of cases. Interestingly, the OTS was calculated for each patient by adding the determined number of variables (VA, rupture, endophthalmitis, perforating or penetrating injury, retinal detachment and afferent pupillary defect) at presentation and converted to 5 OTS categories, as performed in the OTS study. Then, the final VA of their study groups was compared to those in the OTS study group. No statistically significant difference was observed between the categorical distribution of their patients and those in the OTS study, validating the OTS predictive value in OS. Therefore, a higher OTS category correlates with a better prognosis, so OTS could be considered a prognostic factor when counselling patients [8].

## Conclusion

OS may develop a few days until several years after the trauma. The rate of OS development depends on several factors, such as the IOFB dimensions, shape and composition; IOFB localization; and associated trauma tissue reaction.

OS clinical signs are multiple, ranging from iron deposits on the corneal layers, heterochromia, dilated and nonreactive pupil to retinal pigmentary degeneration.

A complete ophthalmologic examination is mandatory, with an accurate review of both the anterior and posterior segments. In the case of an undetected IOFB, CT is considered the gold standard, but ultrasonography can also play an important role.

Ferrous IOFBs should also be removed if OS is already diagnosed because VA, ocular signs and ERG may improve. However, if the IOFB is located in the lens or subretina and OS is not yet developed, follow-up can be an option, with VA assessment, ophthalmic evaluation and ERG, because *b*-wave amplitude reduction arises earlier than clinical signs.

Treatment depends on the IOFB location, whether it is embedded in the retina and how clear the lens is. Regarding the anterior segment, IOFBs can be removed with IOM or forceps through limbal paracentesis. Regarding the posterior segment, after patient counselling, PPV can be performed, removing the IOFB with IOM or forceps through an enlarged sclerotomy or the limbus.

Despite clinical improvement, IOFBs continue to be overlooked, and OS develops, mainly due to delayed presentation or missed diagnosis. Therefore, it is mandatory that the use of protective glasses be recommended. Finally, spreading knowledge about ocular trauma and OS further benefits the care of patients.
